# Altered Topological Properties of Brain Structural Covariance Networks in Patients With Cervical Spondylotic Myelopathy

**DOI:** 10.3389/fnhum.2020.00364

**Published:** 2020-09-04

**Authors:** Cuili Kuang, Yunfei Zha, Changsheng Liu, Jun Chen

**Affiliations:** Department of Radiology, Renmin Hospital of Wuhan University, Wuhan, China

**Keywords:** cervical spondylotic myelopathy, brain structural covariance network, graph theory analysis, small world, topological properties

## Abstract

**Background:**

Brain structural alterations play an important role in patients with cervical spondylotic myelopathy (CSM). However, while there have been studies on regional brain structural alterations, only few studies have focused on the topological organization of the brain structural covariance network. This work aimed to describe the structural covariance network architecture alterations that are possibly linked to cortex reorganization in patients with CSM.

**Methods:**

High-resolution anatomical images of 31 CSM patients and 31 healthy controls (HCs) were included in the study. The images were acquired using a sagittal three-dimensional T1-weighted BRAVO sequence. Firstly, the gray matter volume of 90 brain regions of automated anatomical labeling atlas were computed using a VBM toolbox based on the DARTEL algorithm. Then, the brain structural covariance network was constructed by thresholding the gray matter volume correlation matrices. Subsequently, the network measures and nodal property were calculated based on graph theory. Finally, the differences in the network metrics and nodal property between groups were compared using a non-parametric test.

**Results:**

Patients with CSM showed larger global efficiency and smaller local efficiency, clustering coefficient, characteristic path length, and sigma values than HCs. Patients with CSM had greater betweenness in the left superior parietal gyrus (SPG.L) and the left supplementary motor area (SMA.L) than HCs. Besides, patients with CSM had smaller betweenness in right middle occipital gyrus. The brain structural covariance networks of CSM patients exhibited equal resilience to random failure as those of HCs. However, the maximum relative size of giant connected components was approximately 10% larger in HCs than in CSM patients, upon removal of 44 nodes in targeted attack.

**Conclusion:**

These observed alternations in global network measures in CSM patients reflect that the brain structural covariance network in CSM exhibits the less optimal small-world model compared to that in HCs. Increased betweenness in SPG.L and SMA.L seems to be related to cortex reorganization to recover multiple sensory functions after spinal cord injury in CSM patients. The network resilience of patients with CSM exhibiting a relative mild vulnerability, compared to HCs, is probably attributable to the balance and interplay between cortex reorganization and ongoing degeneration.

## Introduction

Cervical spondylotic myelopathy (CSM) is a common spinal cord dysfunction that causes motor and sensory deficits of the limbs, neck pain, and cervical vertigo with gait instability (Yarbrough et al., 2012). Usually, CSM is regarded as a specific form of incomplete spinal cord injury (SCI) (Sharp and Simon, 2005). Many brain studies have reported functional and structural changes in the cortex of patients with CSM, and brain reorganization after SCI has been accepted as the pivotal factor affecting the function and rehabilitation of patients with CSM (Zhou et al., 2014; Chen et al., 2019). Cortical damage or plasticity in the central nervous system has also been suggested to influence the clinical symptoms, manifestations, and functional rehabilitation of patients with CSM (Zhou et al., 2014). Specifically, functional magnetic resonance imaging (MRI) studies have reported increased volume of activation within the primary motor cortex and decreased volume of activation within the primary sensory cortex in patients with CSM, followed by cortical reorganization after decompressive surgery (Dong et al., 2008; Holly, 2009; Duggal et al., 2010). A magnetic resonance spectroscopy study detected decreased *N*-acetylaspartate/creatine, which implied axonal or neuronal damage/loss in the motor cortex of patients with CSM, and the presence of neurological damage was suggested to be linked to axonal and/or neuronal injury (Kowalczyk et al., 2012). Furthermore, a recent study has reported decreased gray matter volume (GMV) in the left superior parietal lobule and decreased white matter (WM) volume in the right temporal lobe, occipital lobule, and calcarine gyrus in patients with subacute incomplete cervical cord injury (Chen et al., 2019). Alterations in the central nervous system not only occur in incomplete SCI, other brain studies focusing on patients with complete SCI have also observed gray matter alterations. A previous voxel-based morphometry (VBM) study detected significant gray matter atrophy in the primary motor cortex, primary somatosensory cortex, supplementary motor area, and thalamus during the early stage of SCI in humans (Hou et al., 2014). Another study found decreased GMV in the anterior cingulate cortex, left insula, left secondary somatosensory cortex, and bilateral thalamus in patients with chronic traumatic SCI (Jutzeler et al., 2016). Importantly, a previous study reported that progressive atrophic and microstructural changes across the sensory system were closely related to sensory outcome in patients with SCI (Grabher et al., 2015). These findings suggest that structural alterations play a key role in patients with incomplete or complete SCI. However, while these studies have provided important information on regional brain structural alterations, only few have focused on the topological organization of the brain structural covariance network.

Nowadays, brain structural covariance network analysis relying on morphological metrics such as the GMV and cortical thickness is widely applied in neuropathological mechanism researches (He et al., 2007; Wen et al., 2011). Based on the statistical correlations of the morphological descriptors and the graph theory method, network analysis can explore the topological organization of the whole brain, thus providing comprehensive network-level information (He et al., 2007; Lv et al., 2010). This type of information extends and complements conventional brain structural MRI findings. The biological basis of structural covariance network analyses is the interregional correlations of gray matter volumes or thicknesses, which are considered to represent brain regional connectivity of the network (Sone et al., 2019). This method can extract measures of inter-regional connectivity from the covariance patterns of gray matter volumes or thicknesses and may provide more knowledge on neuropsychiatric diseases when used in combination with functional or diffusion imaging (Alexander-Bloch et al., 2013; Evans, 2013; Sone et al., 2019). In large-scale complex human brain network analysis, this type of brain structural covariance network possesses small-world properties and the other topological properties, such as network centrality and resilience. A small-world network with extensive local clustering and short path lengths makes it an attractive model for information processing through minimal wiring costs (Sporns et al., 2005; Achard and Bullmore, 2007). In other words, a small-world network processes high global efficiency reflecting the network’s capacity for parallel information transfer and high local efficiency indicating the information transfer between adjacent nodes. The nodal betweenness is commonly considered as the nodal centrality and quantifies the influence of a node in connecting other nodes in a network (Freeman, 1977; Cheng et al., 2015). A node with high betweenness is thus crucial to efficient communication and can reach the other nodes on short paths (Barthélemy, 2004). The betweenness equals the fraction of all shortest paths in the network that pass through a given node (Rubinov and Sporns, 2010; Cheng et al., 2015) and reflects the dynamic performances of the network, such as the speed of information transfer (He et al., 2008). Resilience indicates the tolerance of the network against random or targeted attack, and is relevant to the stability of the network (Achard et al., 2006; He et al., 2008). Various neurological diseases are characterized by the topological properties of the brain structural covariance network (Yao et al., 2010; Shi et al., 2012; Tao et al., 2018; Zou et al., 2018; Liu et al., 2019), such as the small-world property, network centrality, and resilience (He et al., 2008, 2009).

In this study, prior to constructing a brain structural covariance network, we used a VBM toolbox based on the diffeomorphic anatomical registration through exponentiated lie algebra (DARTEL) algorithm to obtain the precise GMV as the network morphological descriptor (Ashburner, 2007) and constructed the brain structure covariance network using the graph analysis toolbox (GAT) (Hosseini et al., 2012). Next, we calculated and compared global and local efficiency, small-world parameters, nodal betweenness, and resilience of the brain structural covariance networks of the patients with CSM and healthy controls (HCs). We hypothesized that the changes in the patterns of the network properties evaluated in the patients with CSM will correspond to the patterns of cortex reorganization, which has been reported in previous studies, and we expected the existence of a potential link between the alternation of network properties and cortex organization in patients with CSM.

## Materials and Methods

### Participants

A total of 33 right-handed patients with CSM [17 women and 16 men; age range, 42–67 years; mean age ± standard deviation (SD), 54.78 ± 8.41 years] were recruited at the Renmin Hospital of Wuhan University through convenience sampling. The mean duration of symptoms from disease onset to the date of MRI examination was 37.0 ± 25.1 months (range, 3 months to 8 years). All the patients met the following inclusion criteria: (1) cervical spondylosis, (2) an ossified posterior longitudinal ligament, and (3) demyelination with hyperintensity of cord on T2-weighted imaging (Zhou et al., 2014). Two radiologists determined the spinal cord compression level at which the cord surface was clearly indented, or cord diameter was narrowed by compression. The exclusion criteria were as follows: trauma- or infection-related cord compression, any other neurological disorder, a history of decompression surgery, and any contraindications for MRI. The clinical severity of myelopathy was assessed using the Japanese Orthopaedic Association (JOA) score system (Yonenobu et al., 2001) and the neck disability index (NDI) questionnaire. The JOA score system evaluates the severity of myelopathy by assigning scores based on the degree of dysfunction, and the NDI questionnaire measures the activities of daily living in patients with neck pain. As HCs, 33 right-handed age- and sex-matched healthy volunteers (18 women and 15 men; age range, 40–63 years; mean age ± SD, 53.52 ± 8.13 years) were recruited via community health screenings. Two patients with CSM and two HCs were excluded due to obvious blur artifacts observed in the structural images. Finally, 31 patients with CSM and 31 HCs were included in the analyses. The current study was approved by the ethics committee for clinical research of Renmin Hospital of Wuhan University and was conducted in accordance with the principles of the Declaration of Helsinki. All the participants gave written informed consent.

### MRI Procedure

A GE Discovery 750 3.0-Tesla MR scanner equipped with an eight-channel head coil was used to acquire the MR images. High-resolution anatomical images were acquired using a sagittal three-dimensional T1-weighted BRAVO sequence with the following parameters: repetition time/echo time = 7.2/2.7 ms, inversion time = 450 ms, flip angle = 12°, number of slices = 160, slice thickness = 1.0 mm, field of view = 25.6 cm × 25.6 cm, readout bandwidth = 41.67 kHz, and in-plane matrix = 256 × 256. Sagittal and axial conventional T1-weighted, T2-weighted, and T2 fluid-attenuated inversion recovery images of the brain and cervical spinal cord were acquired for patient diagnosis. For the two groups, the time period of data acquisition was from March 2017 to May 2018.

### Data Preprocessing

#### Measurements of the GMV

To obtain the GMV, we used a VBM8 toolbox based on the DARTEL algorithm (Ashburner, 2007). VBM8 was performed using statistical parametric mapping (SPM8)^1^ running on MATLAB (R2013a, Mathworks, Natick, MA, United States). First, each subject’s structural MR images were segmented into gray matter (GM), WM, and cerebrospinal fluid in the native-space using the segmentation module in SPM8 (Ashburner and Friston, 2005). Secondly, the study-specific DARTEL templates (GM, WM) were generated from the entire image dataset using the DARTEL technique (Ashburner, 2007). Thirdly, the DARTEL templates were affine registered to the tissue probability maps in the Montreal Neurological Institute (MNI) space. Then non-linear warping of the native-space segmented images was normalized to match the DARTEL templates (GM, WM) in the MNI space, during this procedure, images were modulated to ensure that relative volumes of GM and WM were preserved following the spatial normalization. Finally, the modulated and normalized images were smoothed with a 6-mm full-width at half-maximum isotropic gaussian kernel.

#### Construction of the Brain Structural Covariance Network

**FIGURE 1:**
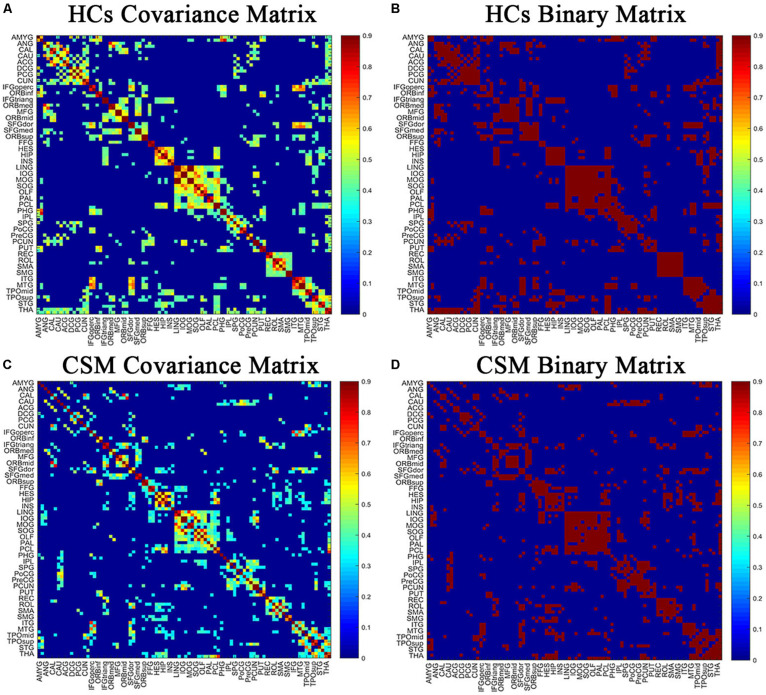
The correlation matrices of healthy controls (HCs) **(A,B)** and patients with cervical spondylotic myelopathy (CSM) groups **(C,D)** (left, thresholded; right, binarized). The color bar indicates the strength of the Pearson correlation coefficients between brain areas. The *X*/*Y* axes represent the 90 ROIs from the AAL atlas, with the exception of the 26 cerebellar regions. For clarity, brain regions of AAL atlas without distinction between left and right are labeled. For the full forms of the abbreviations of the brain regions, see Table 1.

Pearson correlation coefficients between smoothed, modulated, and normalized GMVs were calculated across all participants for each group to generate N × N interregional correlation matrices using the GAT (Hosseini et al., 2012; Figures 1A,C; binarized, Figures 1B,D) (N represents the number of nodes). We selected 90 regions of interest of the automatic anatomic labeling (AAL) atlas as nodes (Tzourio-Mazoyer et al., 2002). A linear regression analysis was performed to remove the influence of nuisance covariates, namely age, sex, and total intracranial volume, in each group. Then, the interregional correlation matrices were thresholded across a range of network densities (0.1 to 0.5 with an interval of 0.02). The minimum density guaranteed that the network for each of the two groups was fully connected. The maximum density prevented the networks from exhibiting randomness. Previous studies have indicated that a maximum network density should be below 50% since structural networks with more than 50% connections are likely non-biological (Kaiser and Hilgetag, 2006; Hosseini et al., 2012). Lastly, network topologies were calculated and compared between the two groups at each density.

#### Graph Theory Analysis

Clustering coefficient (Cp) and characteristic path length (Lp) are two crucial metrics of the complex network (Bullmore and Sporn, 2009; Rubinov and Sporns, 2010). Cp is the average clustering coefficient across all nodes in a network and estimates the functional segregation for specialized processing to occur within densely interconnected groups of brain regions (Watts and Strogatz, 1998; Rubinov and Sporns, 2010). Lp is the average shortest path length between all pairs of nodes in the network and is the most commonly used measure of functional integration between brain regions (Watts and Strogatz, 1998; Rubinov and Sporns, 2010). To characterize the topological properties of the brain network, the two metrics are compared to the null random networks (20 generated null networks). Thereafter, the normalized Cp (γ = Cp/Cp_rand_), the normalized Lp (λ = Lp/Lp_rand_), and the small-world index Sigma (σ = γ/λ) can be obtained. If γ > 1, and λ = ∼1 or σ > 1, the brain network possesses small-world properties. This implies that the clustering coefficient of the brain structural network is significantly higher than that of random networks, and the characteristic path length is comparable to that of random networks (Bullmore and Sporn, 2009; Rubinov and Sporns, 2010). Global efficiency is inversely related to average minimum path length and measures the network’s capacity for parallel information transfer between nodes via multiple series of edges (Achard and Bullmore, 2007). Local efficiency measures the information transfer in the immediate neighborhoods of each node and indicates the fault tolerance of the network to deletion of individual nodes (Achard and Bullmore, 2007).

For regional nodal characteristics of the brain structural covariance network, we discussed and measured the normalized nodal betweenness (nodal betweenness normalized by the average betweenness of the network). The normalized nodal betweenness captures the influence of a node over information flow between other nodes in the network (He et al., 2008). The network metrics, small-world parameter, and normalized nodal betweenness were calculated using the GAT.

In previous studies, resilience have been investigated in brain networks (Achard et al., 2006; Kaiser et al., 2007). Resilience indicates the tolerance of the brain network to random failure or targeted attack. Random failure could be simulated by removing one node from the network and targeted attack could be evaluated by removing nodes in the order of decreasing degree or betweenness. These processes can be repeated until all of the nodes are removed. The resilience can be visualized by plotting the size of the giant connected components as a function of the number of nodes removed (Achard et al., 2006). The relative size of the giant connected component represents the size of the giant connected component normalized by the biggest connected component of the network before the nodes are removed. In the present study, we first randomly removed a node from the brain networks of the two groups and measured the relative size of the giant connected component. We repeated the process until all the nodes were removed. Subsequently, we removed nodes in order of decreasing betweenness. Similarly, the process was repeated until all the nodes were removed. Lastly, we obtained the curves describing the changes in the relative size of the giant connected component as a function of the number of nodes removed.

### Statistical Analyses

The distribution of age and sex between groups was assessed using the two-sample *t*-test and the chi-squared test, respectively. The differences in network measures between the two groups were tested using a non-parametric permutation algorithm (Bullmore et al., 1999; He et al., 2008; Liu et al., 2016; Zou et al., 2018). First, we calculated the network metrics (Cp, Lp), small-world parameter (Sigma), and nodal characteristic (nodal betweenness) separately for the two groups across the density range (0.1:0.02:0.5). Next, we randomly rearranged each participant’s set of modulated GMV data to one or the other of the two groups and computed the correlation matrix for each randomized group, after which we calculated the network metrics and nodal characteristic for each randomized group across the same density range (0.1:0.02:0.5) as in the real brain networks. The randomization procedure was repeated 1000 times. Lastly, we employed the 95 percentile points of each distribution as the critical values for a one-tailed test of the null hypothesis with a type I error probability of 0.05 (He et al., 2008) and extracted the curves that presented the difference in network metrics (between two groups) as functions of network density.

Subsequently, in order to reduce the impact of thresholding process, we compared the curve differences between groups through a summary measure using areas under a curve (AUC) analysis. To test the significance of the between-group differences in AUC of each network measure, a similar approach of a non-parametric permutation algorithm was applied (Hosseini et al., 2012). First, the real between-group difference in AUC for each network measure was calculated. Then, for each randomized group, the between-group difference in AUC for each network measure was computed for the 1000 times of randomization procedure. Lastly, the 95 percentile points of each distribution as the critical values for a one-tailed test of the null hypothesis with a type I error probability of 0.05 was used.

To test whether the two group networks behaved differently against random failure and targeted attack, we applied a permutation analysis following the procedure mentioned for analyzing between-group differences in network measures, which has been performed in a previous study (Hosseini et al., 2012). First, the real between-group difference in network resilience against random failure and targeted attack by the deletion of nodes were calculated. Next, for each randomized group, the between-group difference in network resilience against random failure and targeted attack by the deletion of nodes (as calculated in the real brain networks) were computed. Lastly, the 95 percentile points of each distribution as the critical values for a one-tailed test of the null hypothesis with a type I error probability of 0.05 was used.

## Results

### Demographic and Clinical Data Profiling

Table 2 summarizes the demographic and clinical data of patients with CSM and HCs. There were no significant differences in age (*P* = 0.752) and sex (*P* = 0.95) between the two groups. However, JOA scores, NDI scores, and duration of symptoms were significantly different between the two groups (*P* < 0.0001).

**TABLE 1:** Abbreviations of brain regions of AAL atlas (without distinction between left and right).

Abbreviation	AAL region	Abbreviations	AAL regions
AMYG	Amygdala	MOG	Middle occipital gyrus
ANG	Angular gyrus	SOG	Superior occipital gyrus
CAL	Calcarine cortex	OLF	Olfactory cortex
CAU	Caudate nucleus	PAL	Lenticular nucleus, pallidum
ACG	Anterior cingulate gyrus	PCL	Paracentral lobule
DCG	Middle cingulate gyrus	PHG	Parahippocampal gyrus
PCG	Posterior cingulate gyrus	IPL	Inferior parietal lobule
CUN	Cuneus	SPG	Superior parietal gyrus
IFGoperc	Inferior frontal gyrus (opercular)	PoCG	Postcentral gyrus
ORBinf	Inferior frontal gyrus (inferior)	PreCG	Precentral gyrus
IFGtriang	Inferior frontal gyrus (triangular)	PCUN	Precuneus
ORBmed	Orbitofrontal cortex (medial)	PUT	Lenticular nucleus, putamen
MFG	Middle frontal gyrus	REC	Rectus gyrus
ORBmid	Orbitofrontal cortex (middle)	ROL	Rolandic operculum
SFGdor	Superior frontal gyrus (dorsal)	SMA	Supplementary motor area
SFGmed	Superior frontal gyrus (medial)	SMG	Supramarginal gyrus
ORBsup	Orbitofrontal cortex (superior)	ITG	Inferior temporal gyrus
FFG	Fusiform gyrus	MTG	Middle temporal gyrus
HES	Heschl gyrus	TPOmid	Temporal pole (middle) gyrus
HIP	Hippocampus	TPOsup	Temporal pole (superior) gyrus
INS	Insula	STG	Superior temporal gyrus
LING	Lingual gyrus	THA	Thalamus
IOG	Inferior occipital gyrus		

**TABLE 2:** Demographic and clinical data of patients with CSM and HCs.

Subject	CSM group	HCs group	*P*-value
N	31	31	n/a
Age	54.78 ± 8.41	53.52 ± 8.13	0.752
Gender (male/female)	16/17	15/18	0.95
Handedness (right/left)	31/0	31/0	n/a
Laterality of spinal cord compression (right/left/bilateral)	17/12/4	n/a	n/a
JOA scores	10.53 ± 2.57	17 ± 0	<0.0001
NDI scores	0.345 ± 0.102	0.009 ± 0.001	<0.0001
Duration of symptoms (month)	37.0 ± 25.1	n/a	n/a

*CSM, cervical spondylotic myelopathy; HCs, healthy controls; JOA, Japanese Orthopaedic Association; NDI, neck disability index.*

### Network Analyses

#### Global Network Measures and Small-World Parameter

**FIGURE 2:**
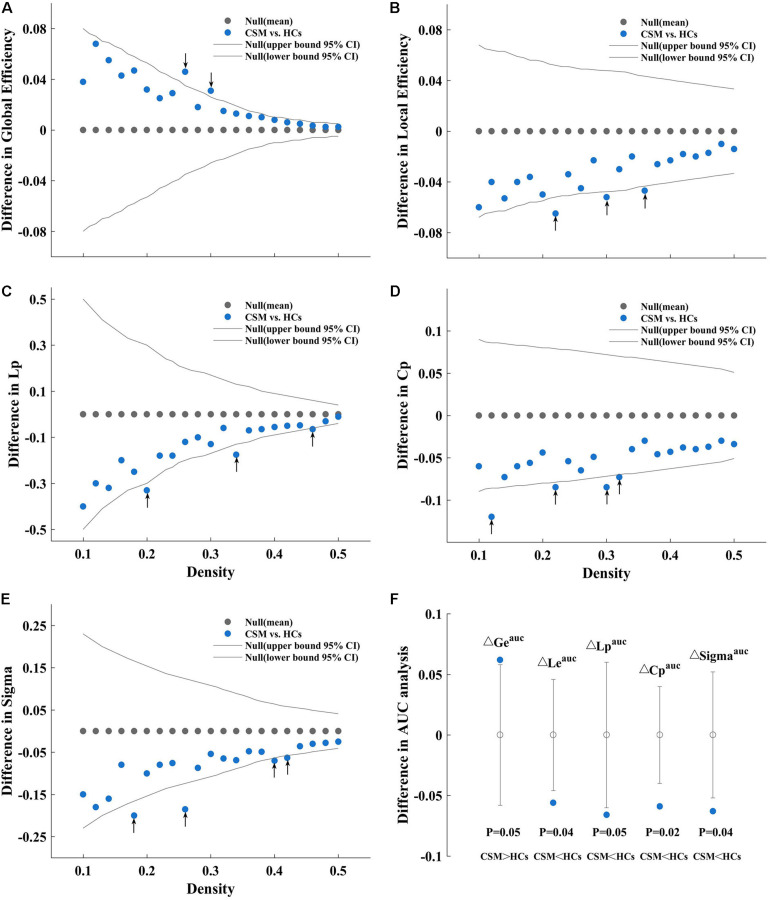
Between-group differences in global efficiency **(A)**, local efficiency **(B)**, characteristic path length (Lp) **(C)**, clustering coefficient (Cp) **(D)**, and Sigma **(E)** as a function of density. Between-group differences in areas under the global efficiency (ΔGe^auc^), local efficiency (ΔLe^auc^), Lp (ΔCp^auc^), Cp (ΔLp^auc^), and Sigma (ΔSigma^auc^) curves **(F)**, from left to right sequentially). As an example, we have described **(A)**. **(A)** Denotes the differences (azure dots) in the global efficiency between the patients with cervical spondylotic myelopathy (CSM) and the healthy controls (HCs) as a function of density thresholds. The gray dots represent the mean values and the gray lines represent the 95% confidence intervals of the between-group differences obtained 1000 permutation tests at each density value. The black arrows indicate significant difference in global efficiency between the two groups (*P* < 0.05). Patients with CSM show larger global efficiency in the brain structural covariance networks than HCs at thresholds 0.26 and 0.30. Similarly, **(B)** denotes that patients with CSM show smaller local efficiency in the brain structural covariance networks than HCs at threshold 0.22, 0.30, and 0.36. **(C)** Denotes that patients with CSM show smaller Lp in the brain structural covariance networks than HCs at threshold 0.20, 0.34, and 0.46. **(D)** Denotes that patients with CSM show smaller Cp in the brain structural covariance networks than HCs at threshold 0.12, 0.22, 0.30, and 0.32. **(E)** Denotes that patients with CSM show smaller Sigma in the brain structural covariance networks than HCs at thresholds 0.18, 0.26, 0.40, and 0.42. **(F)** Denotes that patients with CSM show significantly larger Ge^auc^ (*P* = 0.05) and smaller Le^auc^ (*P* = 0.04), Lp^auc^ (*P* = 0.05), Cp^auc^ (*P* = 0.02), and Sigma^auc^ (*P* = 0.04) in the brain structural covariance networks than HCs in the areas under curve (AUC) analysis.

In the current study, we investigated the between-group differences in global network measures and small-world parameter on brain structural covariance network at a range of densities (0.1:0.02:0.5). Compared with HCs, the network of patients with CSM showed greater global efficiency (Figure 2A) and smaller local efficiency (Figure 2B), Lp (Figure 2C), Cp (Figure 2D), and Sigma (Figure 2E) at several densities across the range (*P* < 0.05). In addition to comparing the networks at various densities, we compared the AUC for global network measures and small-world parameter curves (across the density range of 0.1:0.02:0.5) between the two groups. We observed that the network of patients with CSM had significantly larger AUC for global efficiency (*P* = 0.05) and smaller AUC for local efficiency (*P* = 0.04), Lp (*P* = 0.05), Cp (*P* = 0.02), and Sigma (*P* = 0.04) (Figure 2F).

#### Nodal Betweenness

**FIGURE 3:**
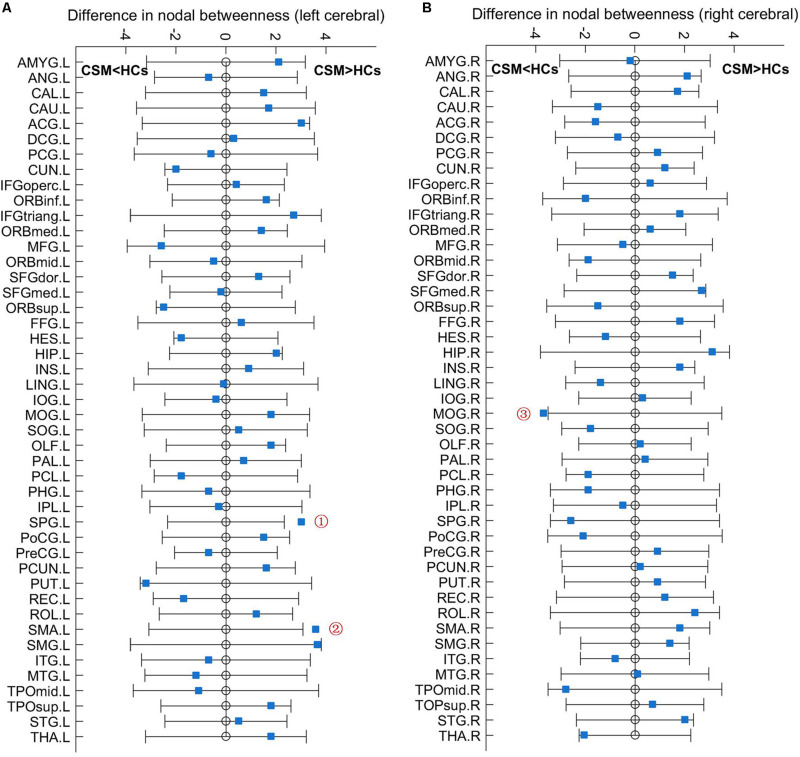
Between-group differences in nodal betweenness in 45 left cerebral regions **(A)** and 45 right cerebral regions **(B)** of AAL atlas. The open circles represent the mean values and the gray bar lines represent the 95% confidence intervals of the between-group differences obtained 1000 permutation tests. Patients with CSM had significantly increased betweenness in the left superior parietal gyrus (SPG.L, indicated as ➀ in panel **A**) and left supplementary motor area (SMA.L, indicated as ➁ in panel **A**) and significantly decreased betweenness in the right middle occipital gyrus (MOG.R, indicated as ➂ in panel **B**) compared to HCs. For the full forms of the abbreviations used to denote the brain regions, see Table 1.

**FIGURE 4:**
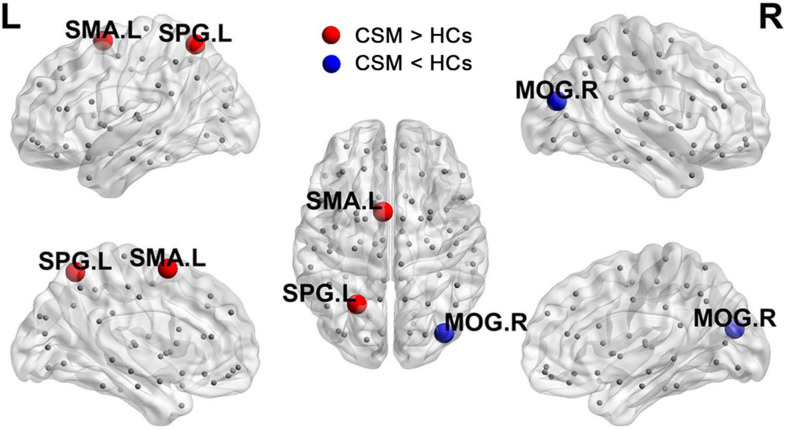
Brain regions with significant changes in nodal betweenness in patients with CSM compared to HCs.

Figure 3 shows the differences (azure dots) in nodal betweenness in 45 left cerebral regions (Figure 3A) and 45 right cerebral regions (Figure 3B) of AAL atlas between patients with CSM and HCs. Table 3 lists the brain regions with significant changes in nodal betweenness in patients with CSM compared with that in HCs. Figure 4 highlights these brain regions in anatomical space. The red (blue) nodes indicate increased (decreased) nodal betweenness in the CSM group compared to that in the HCs group.

**TABLE 3:** AUC analysis for nodal betweenness in patients with CSM and HCs.

Relationship	AAL region	CSM betweenness	HCs betweenness	*P*-value
CSM > HCs	Left superior parietal gyrus	3.63	0.28	0.01
	Left supplementary motor area	2.59	0.46	0.03
CSM < HCs	Right middle occipital gyrus	0.07	0.52	0.05

*CSM, cervical spondylotic myelopathy; HCs, healthy controls; AUC, areas under the curve; AAL, automatic anatomic labeling.*

#### Network Resilience

**FIGURE 5:**
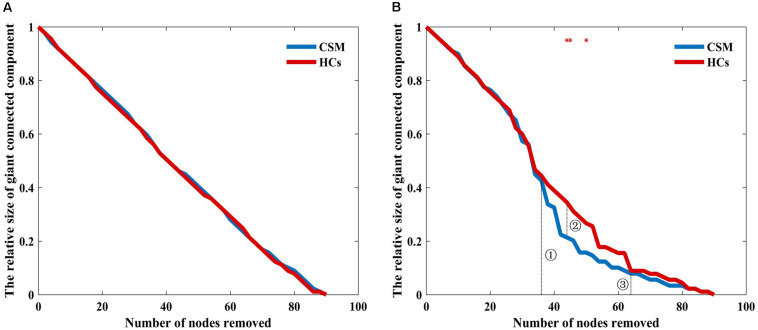
Relative size of the giant connected component as a function of the number of nodes removed by random failure **(A)** and targeted attack **(B)** in the two groups. Red stars show where the difference in the relative size of the giant connected component between the groups is statistical significant (*P* < 0.05).

Figure 5A shows that the brain structural covariance network of the CSM group was as resilient to random failure as that of the HCs group. However, in targeted attack, when the number of nodes removed were less than 37 (Figure 5B, dotted line indicated with ➀) or more than 63 (Figure 5B, dotted line indicated with ➂), the relative size of giant connected components of the two groups were equivalent. When the number of nodes removed were in the range of 37 to 63, the maximum difference of the relative size of giant connected components between the two groups was nearly 10% when 44 nodes were removed (Figure 5B, dotted line indicated with ➁). The difference in the relative sizes of giant connected components between the two groups was significant when 44, 45, and 50 nodes were removed (where the red stars indicate in Figure 5B).

## Discussion

Here, we used graph theory-based network analyses to compare the topological properties of the brain structural covariance networks between patients with CSM and HCs. We found that patients with CSM had larger global efficiency and smaller local efficiency, Lp, Cp, and Sigma values than HCs. Patients with CSM had increased nodal betweenness in the SMA.L, SPG.L, and decreased betweenness in the MOG.R compared to that in the HCs. Both groups exhibited equal resilience to random failure. When 44 nodes were removed in the analysis for targeted attack, the maximum relative size of giant connected components was approximately 10% larger in HCs than in patients with CSM. Further, the difference in the relative size of giant connected components between the two groups was significant upon removal of several different number of nodes.

### Altered Global Network Measures and Small-World Parameter in Patients With CSM

The small-world network reflects an attractive model for the combination of high clustering and short path length, which confers a capability for both specialized or modular processing in local neighborhoods and distributed or integrated processing over the entire network (Achard et al., 2006). In the present study, the network measures Cp, Lp, and Sigma in the brain structural covariance network of patients with CSM were significantly smaller than those of HCs. These results suggest that the brain structural covariance network of CSM patient exhibits an unoptimizable topological organization with lower Cp and shorter Lp. In addition, we also observed larger global efficiency and smaller local efficiency in the brain structural covariance network of patients with CSM compared to that of HCs. Global efficiency is inversely related to Lp and measures the capacity of information transfer between nodes by multiple parallel paths (Achard and Bullmore, 2007). Decrease in Lp indicates the enhanced ability for serial information transfer between remote regions of the brain (Rubinov and Sporns, 2010). Increase in global efficiency indicates the intensive capacity for parallel information transfer between brain regions via multiple series of paths. Previous studies have demonstrated that the clustering coefficient can be regarded as a measure of the local efficiency of information transfer (Achard and Bullmore, 2007). Decreases in local efficiency and Cp indicate the inefficient information transfer in the immediate neighborhoods of each node and a weaker local specialization and modular processing in adjacent neighborhoods (Rubinov and Sporns, 2010). Studies have demonstrated that cortical reorganization is an innate attribute of patients with CSM to compensate for motor damage after SCI (Tan et al., 2015; Bhagavatula et al., 2016). Synaptic plasticity involving modification of pre-existing connections and anatomical plasticity involving the development of new circuitry through sprouting of axons and dendrites are the two main adaptive procedures of cortical reorganization in patients with CSM (Dong et al., 2008). A previous study demonstrated that the brain seems to use available pre-existing neural systems by reducing inhibition in the early recovery stage after SCI. However, during the late stage, the brain appears to enhance the original systems or recruit other systems via neural circuit plasticity (Nishimura and Isa, 2009). It is expected that the cortex reorganization for the development of new circuitry may enhance the information communication and function integration for remote brain regions to recovery sensorimotor function and contribute to the shorter path lengths in the brain structural covariance network of patients with CSM. Although, the lower Cp and shorter Lp in the brain structural covariance network of CSM patients indicate the topological properties deviate from the attractive small-world model compared to HCs. In the subsequent network resilience analysis, the network resilience of patients with CSM was vulnerable compared to that of HCs in the targeted attack. Decrease in local efficiency also reflects the inferior network resilience to the deletion of individual nodes. It is suggested the low network resilience requires intensive capacity for parallel information transfer and communication across remote brain regions to equilibrate for maintaining the basic biological characteristics of the brain structural covariance network of patients with CSM, which seems to response the innate cortex reorganization after SCI in patients with CSM.

### Altered Nodal Betweenness in Patients With CSM

The betweenness of the SPG.L was significantly higher in patients with CSM than in HCs. Recently, a resting-state functional MRI (fMRI) study has reported increased regional homogeneity in the right SPG in patients with CSM (Tan et al., 2015). Another task fMRI study reported enhancement activation in the SPG and inferior parietal lobule following postoperative recovery of function in patients with CSM (Tam et al., 2010). The neuron functional activity alterations in the SPG of patients with CSM were interpreted to integrate and regulate the injury information from the primary sensory cortex, and greater activity coherence was considered to be required in the somatosensory cortex to compensate for the decreased sensory loss (Tan et al., 2015). In addition, a recent study reported GM atrophy in the SPG.L in subacute CSM patients (Chen et al., 2019), which seems to contradict the increase in nodal betweenness in the SPG.L of patients with CSM in our study. We speculated that the inconsistency may result from the different stage of patients with CSM between the former and the present studies. The former study focused on the subacute stage patients with CSM and the current study did not restrict the condition. However, these results indicate the alterations occurring in the SPG.L of patients with CSM. Nodes with high betweenness or degree are often regarded as network hubs (Bullmore and Sporn, 2009). Network hubs and their connections play pivotal roles in information integration and efficient neuronal signal communication in the central nervous system (van den Heuvel and Sporns, 2013). In brain structural covariance networks, edges correspond to axonal or synaptic links that form the biological infrastructure for neuronal signaling and communication (van den Heuvel and Sporns, 2013). The increased betweenness in the SPG.L of patients with CSM in the current study indicates the high fraction of all shortest paths linking the other axonal or synaptic in the network that pass through it (Rubinov and Sporns, 2010; Cheng et al., 2015) and suggests the fast information transfer between it and the other neurons (He et al., 2008). SPG, also called the somatosensory association cortex, is an important part of the somatosensory system by receiving the nearby primary somatosensory cortex fibers (Chen et al., 2019). It is speculated that the increased information flow and fast information communication between the SPG.L and other cortical regions may facilitate the recovery of somatosensory function. However, further study with more empirical data may be helpful to investigate the issue. It is worth noting that we didn’t carry out the network hub identification and distribution analysis in the study. Based on the demonstration of the previous study, we treated the SPG.L as the network hub in the network of the patients with CSM. However, an analysis on nodal betweenness-based hub identification and distribution is worth being implemented in the further study to verify the opinion.

In the current study, we also observed increased betweenness in the SMA.L of patients with CSM. The SMA, together with the pre-supplementary motor area, constitutes the medial part of Brodmann area 6C (Nachev et al., 2008). It is located in the dorsomedial frontal cortex, anatomically anterior to the leg representation of the primary motor cortex (Picard and Strick, 1996, 2001). Through retrograde tracing methods, anatomical studies have shown that the pattern of termination of the SMA corticospinal cells resembles that of primary motor cortex projections (He et al., 1995; Wise, 1996). The SMA cells make direct connections to motor neurons and are related to motor output (Dum and Strick, 1991, 1996). A study on post-surgery patients with CSM reported that improvement in dexterity and finer movements of the upper limbs are associated with recruitment areas other than the premotor cortex, the postcentral gyrus and supplementary motor cortices were also recruited to compensate for the damage in the cervical spinal cord (Bhagavatula et al., 2016). Meanwhile, another study reported that patients with CSM exhibit areas of expanded cortical representation, including the adjacent motor territories, SMA, for the affected limb, compared with healthy participants (Holly et al., 2007). A few neurological functions are preserved in patients with CSM by modification of pre-existing connections and recruitment of new pathways in a process similar to that observed in patients with stroke or cervical myelitis (Holly et al., 2007). At the network level, integrative functions are performed by a specific set of brain regions and their anatomical connections and depend on neural communication of interregional projections in the network (Goldman-Rakic, 1988; Zeki and Shipp, 1988; van den Heuvel and Sporns, 2013). Perfect integrative functions facilitate large-scale patterns of synchronization and information flow between connected elements (Singer, 1993; Brovelli et al., 2004). The dynamic performances of the network can be quantified by nodal betweenness and degree (He et al., 2008). That is, the higher betweenness or degree of a node, the more effective information transformation between that and other nodes. In our study, the increased betweenness of the SMA.L in patients with CSM is in accordance with the previously reported rearrangement and recruitment of connections between the SMA.L and motor cortex neurons (Holly et al., 2007; Bhagavatula et al., 2016), suggesting the inherent ability of the brain to improve the efficiency of information transfer and to adapt to changes in the environment.

Moreover, we also observed that the betweenness in the MOG.R was significantly lower in patients with CSM than in HCs. MOG is associated with visual information processing. A recent resting-state fMRI study has reported that the amplitude of low-frequency fluctuations and regional homogeneity in the occipital lobe are decreased in patients with CSM (Chen et al., 2018). The two resting-state functional measures indicate functional activity oscillations (Zang et al., 2007) and synchronization of neurons (Zang et al., 2004), respectively. The decreases of the two brain functional metrics observed in patients with CSM indicate inactivity of the neurons in the occipital lobe cortex. In addition, another study observed decreased WM volume in the right occipital lobule and calcarine gyrus in patients with subacute incomplete cervical cord injury (Chen et al., 2019). The alterations of nodal properties in the MOG.R in our study are in line with previous functional and structural findings in the occipital lobe of patients with CSM. For most patients with CSM, cervical vertigo is also a general symptom that often causes gait instability (Yarbrough et al., 2012). The decreased betweenness in the MOG.R may be associated with altered visual input and output of the occipital lobe in patients with CSM.

It is worth noting that both the previous studies and our study have observed changes in the SPG.L of patients with CSM; the current study further reported changes in the SMA.L. Previous studies focusing on the ipsilateral hemisphere of patients with CSM have demonstrated that the asymmetrical spinal cord compression or asymmetrical spinothalamic sensory loss may be the main interpretation for the changes observed in the ipsilateral hemisphere of patients with CSM (Dong et al., 2008; Zhou et al., 2014; Tan et al., 2015). Additionally, the inconsistent maturity of inter-hemispheric regions has been regarded as another explanation for the changes observed in the ipsilateral hemisphere of patients with CSM (Dong et al., 2008). Though, further study with advanced techniques such as diffusion tensor imaging and advanced statistical analysis may benefit the clarification of the issue.

### Network Resilience in the Two Groups

In network resilience analysis, both the groups including the patients with CSM and the HCs showed equal resilience to random failure. When the number of nodes removed in the targeted attack was in the range of 37 to 60, the maximum relative size of giant connected components was larger in HCs than in patients with CSM by approximately 10%, upon removal of 44 nodes. A previous similar study (He et al., 2008) reported similar resilience to random failure in both patients with Alzheimer’s disease and HCs. However, when 25% core nodes were removed in the targeted attack, the relative size of giant connected components of the Alzheimer’s disease group was reduced by nearly 20% compared with that of HCs, implying vulnerability of the former group to targeted attack. In the present study, the maximum difference of the relative size of giant connected components between the two groups was limited to 10% during the entire targeted attack. Statistically, we observed the network resilience of patients with CSM was significantly vulnerable compared to HCs upon removal of several different number of nodes. However, across the entire removal of nodes in targeted attack, the resilience difference between the two groups in our study was less obvious than that in the previous Alzheimer’s disease study. A previous complete thoracic SCI study demonstrated that the remaining motor fibers within the spinal cord are repaired after SCI, but permanent deficits in motor control may still persist (Wrigley et al., 2008). Ongoing degeneration of the transected corticospinal tract may limit the recovery, and changes occurring in fiber tracts above the injured spinal cord level may also hinder the capacity for full motor recovery following SCI (Wrigley et al., 2008). Thus, despite innate cortex reorganization after SCI, patients with CSM may also present a pathological mechanism analogous to that of patients with complete thoracic SCI. However, further research is needed to confirm this assumption. We speculated that the balance and interplay between cortex reorganization and ongoing degeneration may explain the relative mild vulnerability of patients with CSM.

### Study Limitations

Some limitations of the study need to be acknowledged. First, we constructed the brain structural covariance network based on the GMV morphological descriptor. The combined use of cortical thickness may provide novel insights into the complex network properties of patients with CSM. In addition, a whole brain voxel-wise GM analysis provide fundamental GM alteration evidence which should be supportive for the brain structural covariance network analysis. Secondly, the study only discussed the changes of GM. Performing diffusion tensor imaging analyses of WM fiber tracts may provide additional structural information on cortex reorganization in CSM. Thirdly, the non-parametric permutation test for each network measures at each density was not corrected by multiple comparison. Though, the AUC analysis which alleviates the sensitivity of the between group comparison to the thresholding process should improve the validity of the analysis in the study. Moreover, the topological measures only displayed significant differences between the two groups at several network densities. The statistic results seems to be not very robust, which maybe related to the choice of the anatomical parcellation scheme. We took the 90 brain region of AAL atlas as the nodes to construct the brain structural covariance network in this study. The Harvard-Oxford atlas maybe also worth being adopted in the further brain network studies, which should contribute to improve the validity of the statistic significance (Desikan et al., 2006). Finally, we only assessed the topological features of patients with CSM without decompression surgery. Decompression surgery in CSM may relieve symptoms and facilitate cortex reorganization and subsequent recovery of the essential neurological function (Fawcett et al., 2007). Further researches including patients with CSM before and after decompression surgery could provide more clues about cortex reorganization in CSM.

## Conclusion

Here, we described brain structural covariance network topological properties of patients with CSM. Our main findings were as follows: (1) The observed global network measures alternations in patients with CSM patients reflect that the brain structural covariance network exhibits the less optimal small-world model compared to that in HCs. (2) Increased betweenness in the SPG.L and SMA.L seems to be related to cortex reorganization to recover multiple sensory functions after spinal cord injury in patients with CSM. (3) The network resilience of patients with CSM exhibiting a relatively mild vulnerability compared to that of HCs is probably attributable to the balance and interplay between cortex reorganization and ongoing degeneration. These findings contribute to the understanding of cortex reorganization in CSM from a structural network viewpoint.

## Data Availability Statement

The datasets presented in this article are not readily available because to protect the subjects’ privacy. Requests to access the datasets should be directed to YZ, fskyanteam@126.com.

## Ethics Statement

The studies involving human participants were reviewed and approved by Ethics Committee for Clinical Research of Renmin Hospital of Wuhan University. The patients/participants provided their written informed consent to participate in this study.

## Author Contributions

CK and YZ designed the study, collected and analyzed the data, and interpreted the results of the analysis. CK wrote the manuscript. CL and JC contributed to the discussion and manuscript revision. All authors listed meet the criteria for authorship, read, and approved the final manuscript.

## Conflict of Interest

The authors declare that the research was conducted in the absence of any commercial or financial relationships that could be construed as a potential conflict of interest.

The reviewer LG declared a shared affiliation, though no other collaboration, with the authors to the handling Editor.

## Footnotes

1 http://www.fil.ion.ucl.ac.uk/spm

## Abbreviations

AAL: automatic anatomic labeling
AUC: areas under curve
Cp: clustering coefficient
CSM: cervical spondylotic myelopathy
DARTEL: diffeomorphic anatomical registration through exponentiated lie algebra
fMRI: functional magnetic resonance imaging
GAT: graph analysis toolbox
GM: gray matter
GMV: gray matter volume
HCs: healthy controls
JOA: Japanese Orthopaedic Association
Lp: characteristic path length
MOG.R: right middle occipital gyrus
MOG: middle occipital gyrus
MRI: magnetic resonance imaging
NDI: neck disability index
SCI: spinal cord injury
SD: standard deviation
SMA.L: left supplementary motor area
SMA: supplementary motor area
SPG.L: left superior parietal gyrus
SPG: superior parietal gyrus
SPM8: statistical parametric mapping
VBM: voxel-based morphometry
WM: white matter.
